# Cytokines profile in pure neural leprosy

**DOI:** 10.3389/fimmu.2023.1272471

**Published:** 2023-12-05

**Authors:** Izabela Jardim R. Pitta, Debora Bartzen Moraes Angst, Roberta Olmo Pinheiro, Joyce Soares da Silva Vieira, Clarissa Neves Spitz, Ligia Rocha Andrade, Larissa Bittencourt Carvalho, Mariana Andrea Hacker, Euzenir Nunes Sarno, Marcia Rodrigues Jardim

**Affiliations:** ^1^ Leprosy Laboratory, Oswaldo Cruz Institute, Fiocruz, Rio de Janeiro, Brazil; ^2^ Department of Neurology, Antonio Pedro University Hospital/Fluminense Federal University, Niteroi, Brazil; ^3^ Post-Graduate Program in Neurology, Federal University of the State of Rio de Janeiro, Rio de Janeiro, Rio de Janeiro, Brazil; ^4^ Department of Neurology, Pedro Ernesto University Hospital/Rio de Janeiro State University, Rio de Janeiro, Rio de Janeiro, Brazil

**Keywords:** pure neural leprosy, leprosy neuropathy, cytokine profile, leprosy reactions, leprosy nerve conduction studies

## Abstract

**Introduction:**

Pure Neural Leprosy (PNL) is a form of this long time known disease that affects only the peripheral nervous system. Since it is a rare form of the disease, its pathophisiology is still poorly understood.

**Objective:**

Describe the cytokines profile in patients with PNL.

**Methods:**

30 Patients diagnosed with PNL in the Souza Araujo Outpatient Clinic and with cytokines evaluated were selected. They were evaluated by neurologists and diagnosed after a nerve biopsy. Serum levels of IL-1 β, IL-6, IL-10, IL-17, TNF, CCL-2/MCP-1, IFN-ϒ, CXCL-10/IP-10 and TGF-β were evaluates at the moment of the diagnosis.

**Results:**

Neural thickening was a common clinical finding in this groups of patients. Small and medium sensitive fibers signs and symptoms were present in 92% of the patients and motor involvement in 53%. 43% of patients presented neuropathic pain and no one had neuritis TGF-beta, IL-17, CCl-2 and IP-10. CCL-2 levels were associated with demyelinating patters and IP-10 and IL-1o were associated with axonal patterns at NCS.

**Discussion:**

PNL patients’ cytokine profile appears to be different of other clinical forms of leprosy, with the presence of cytokines described in both tuberculoid and lepromatous leprosy. High levels of CCl-2 may be related to the presence of silent neuritis as well as the presence of IL-10. PNL is unique a form of leprosy, therefore, understanding its immunological profiles essential to better understand the disease itself.

## 1 Introduction

Leprosy is an infectious disease that affects both the skin and peripheral nerves (1). *Mycobacterium leprae*, an intracellular pathogen that infects macrophages and Schwann cells, is the causative agent. Most of the disability related to the disease can be attributed to neuropathy (1–4).

The interaction between the innate and adaptive immune response, environmental factors, and genetic predisposition is responsible for the diverse clinical presentations of leprosy (5, 6). In terms of the host immune response, a strong or diminished cell-mediated immune response to the bacilli may occur, giving rise to the tuberculoid and lepromatous forms of the disease, respectively (7). Borderline groups (borderline tuberculoid, borderline borderline, and borderline lepromatous) lie between the two aforementioned polar groups (7, 8).

The borderline forms are considered immunologically unstable forms of the disease and patients with these forms are more susceptible to leprosy reactions (6). Type 1 leprosy reactions are determined by a cell-mediated immune response and clinically characterized by an inflammatory response in the skin or nerve. Type 2 leprosy reactions are mediated by antibody or immune complexes and clinically characterized by painful nodes and systemic symptoms. Neural damage and neuritis may occur in both types of leprosy reactions (6, 9). It is still unknown whether the damage to the peripheral nerve is caused by the *M. leprae* itself or the inflammatory response that it causes (10). It has been suggested that both mechanisms play a role in the clinical spectrum of the disease (11).

Cytokines are small glycoproteins that mediate the communication between cells of the immune system and play important roles in the immunopathology of leprosy (7).The cytokine profiles of the polar forms are distinct, with a predominance of Th1 cytokines in the tuberculoid pole and Th2 cytokines in the lepromatous pole (2, 12, 13).

The pure neural leprosy (PNL) is a rare form of the disease, where only neurological deficits manifest, without skin lesions (1, 14). PNL pathophysiology is still poorly understood (1, 9, 15). The objective of this study is to describe the cytokine profile found in patients with PNL.

## 2 Methods

The study was conducted in the Souza Araújo Outpatient Clinic and was previously approved by the Research Ethics Committee of the institution. Thirty patients with PNL, as diagnosed by nerve biopsy, and for which the cytokine profile had been evaluated at the moment of diagnosis, were selected and included in the study.

All of the patients were first evaluated by dermatologists who excluded the presence of skin lesions. The patients were then referred to a neurologist and submitted to a neurological examination focused on the peripheral nerve system, as well as nerve conduction studies (NCS) to confirm the presence of peripheral neuropathy. The neurological examination and NCS were conducted as described by Vital et al. (2012) (16). All of the selected patients has multiplex mononeuropathy and were evaluated by neurologists with experience in peripheral neuropathy and screened for other causes of multiplex mononeuropathy. The patient was considered to have neuritis when presented with acute nerve function impairment associated with demyelinating features in the NCS. Patients were considered to have neuropathic pain when presented with pain in the territory of a clinically and neurophisiological affected nerve without nerve tenderness and demyelinating features at NCS.

If leprosy persisted as a differential diagnosis, the patients were submitted to a sensory nerve biopsy to confirm the diagnosis. The diagnostic criteria and methodology used for histopathological analysis were those described by Antunes et al. (2012) (15). All of the selected patients had PNL diagnosis confirmed by the sensory nerve biopsy.

Serum cytokine levels were assessed in samples collected at the moment of the diagnosis and stored at -70°C and evaluated by ELISA as described by Ansgt et al. (2020) (17).

Statistical analysis was performed using the Mann–Whitney test with a significance level of 5%. The Statistical Packagefor the Social Sciences (SPSS) v16.0 for Windows was used. The GraphPad Prism 9.0 were used to design the figures.

## 3 Results

Thirty patients were included in the study, 15 were male and 15 were female. The mean age was 46 years. Two patients (6.7%) had leprosy reactions represented by acute neuritis and 13 patients (43.3%) had neuropathic pain at diagnosis. Upon neurological evaluation, 21 patients (70.0%) had neural thickening. The results of the neurological examinations, NCS, and histopathological findings are given in **Tables 1** and **2**.

**Table 1:** Neurological examination and nerve conduction study (NCS) patterns of the study cohort.

Small sensitive fiber signs	27 (90.0%)
Medium sensitive fibers signs	22 (73.3%)
Large sensitive fibers signs	4 (13.3%)
Weakness	16 (53.3%)
Neural thickening	21 (70.0%)
NCS pattern Axonal Demyelinating Mixed Absent SNAP and CMAP	3 (10.0%) 11 (36.7%) 6 (20.0%) 10 (33.3%)

SNAP, sensory nerve action potential; CMAP, compound muscle action potential.

**Table 2:** Histopathological findings of the study cohort.

Epithelioid granuloma	9 (30.0%)
Inflammatory infiltrate	23 (76.7%)
Fibrosis	22 (73.3%)
AFB	6 (20.0%)
Positive PCR	13 (43.3%)

AFB, acid-fast bacilli; PCR, Polymerase chain reaction.

**Figure 1:**
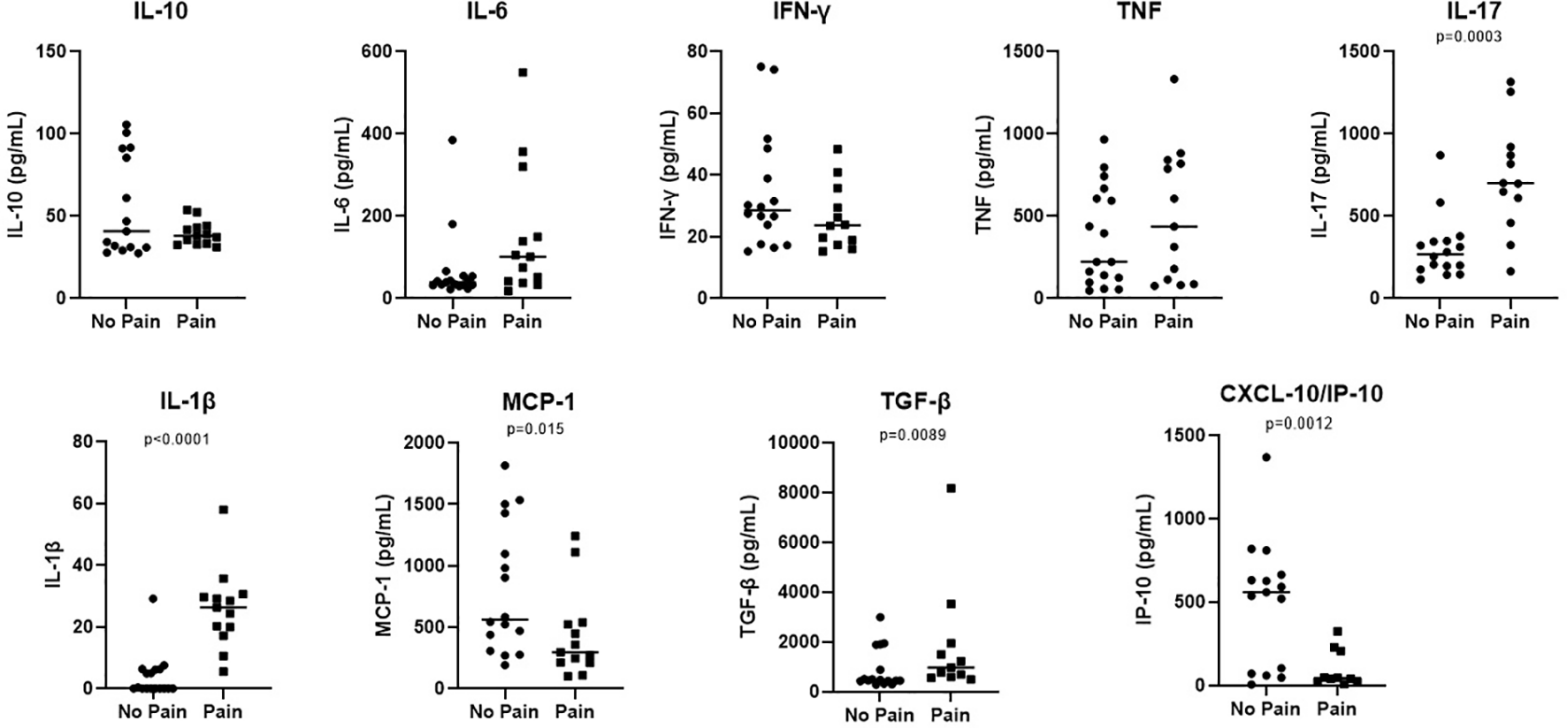
Increased levels of IL-1β and IL-17 are associated with Pain in patients with neural pure form of the disease. Samples from neural pure patients were according the presence or not of pain. Serum levels of IL-10, IL-1β, IL-6, TNF, IL-17, MCP-1, IFN-ϒ, IP-10 and, TGF-β were evaluated by ELISA. The results were showed in a Scatter plot with the median of each group. Significance of the difference between the two groups evaluated were considered when p < 0.05.

The cytokine levels in the serum were evaluated and the medium values of IL-1β and IL-17 were associated with neurophatic pain. The results are shown in **Figure 1**.

Statistically significant correlations between the serum cytokine levels and the clinical, histopathological, or NCS findings are given in **Table 3**.

**Table 3:** Statistically significant differences between the serum cytokine levels and the clinical, histopathological, or nerve conduction study findings of patients with pure neural leprosy.

Cytokine	NCS finding	p-value
MCP-1/CCL2	Demyelinating pattern in mNCS	<0.05
IP-10	Axonal pattern in sNCS	<0.05
IL-10	Axonal pattern in sNCS	<0.05

NCS, nerve conduction studies; mNCS, motor nerve conduction study; sNCS, sensory nerve conduction studies.

## 4 Discussion

PNL is still a poorly understood form of leprosy (9). Cytokines are produced as a response of the immune system to antigens (7) and, although the cytokine profile has been investigated for other forms of leprosy, few studies have evaluated cytokine levels in patients with PNL.

It has been hypothesized that the different forms of leprosy mainly arise owing to factors of the host rather than to variations of the *M. leprae* (2). The classical classification of leprosy was proposed by Ridley and Jopling in 1966 and continues to be used nowadays. It proposes a spectrum with two poles, tuberculoid and lepromatous, and a borderline range in between (8). The cytokine profile is different between the two poles of the disease, with higher levels of IL-7 and IL-15 in the tuberculoid pole and higher IL-4, IL-5, and IL-10 levels in the lepromatous pole (2). Furthermore, high levels of IL-17 have been found in patients of the tuberculoid pole and with leprosy reactions (2). Our patients had high serum levels of both IL-17, which has been related to limited leprosy, and TGF-β, which has been related to disseminated leprosy (2). This indicates that PNL could be a unique form of the disease. As PNL does not fit any of the Ridley–Jopling classifications, it is likely that the cytokine profile of patients with this form may also differ from those with the other clinical forms.

Monocyte chemoattractant protein-1 (MCP-1/CCL2) is a chemokine that regulates the migration and infiltration of macrophages (18). Macrophages are considered one of the major sources of MCP-1/CCL2. Demyelinating patterns in NCS can be suggestive of acute neuritis as well as the beginning of the infection (19).MCP-1/CCL2 was found in high levels in patients with PNL and was also significantly associated with demyelinating patterns in NCS. Most of the patients of our cohort did not have clinical neuritis at diagnosis. Furthermore, silent neuritis has been proposed to play an important role in PNL (9). These data suggest that the function of macrophages may be associated with the beginning of the infection and clinical or silent neuritis.

It has been proposed that *M. leprae* induces Schwann cell death by a pathway that involves proinflammatory cytokines, and TNF-α has already been identified in the nerve biopsies of patients with neuritis (10). TNF-α has been related to demyelination and axonal degeneration as well as immune cell recruitment to the injury site and, in leprosy, it has been detected in reactional states (3). Teles et al. (2007) reported that the levels of TNF-α were higher in patients with PNL especially when AFB were found in the nerve biopsy (20). Although the patients of our cohort did not have clinical neuritis and most of them did not have reactional episodes, the presence of TNF-α in the serum may suggest that it plays a role in the pathogenesis of leprosy neuropathy.

IP-10/CXCL10 has been linked to inflammatory processes, including viral and bacterial infectious diseases (21). Levels of this cytokine were significantly associated with axonal damage in the present study, suggesting that an inflammatory process is involved in the nerve damage observed in PNL. In our sample, levels of this cytokine were also associated with neural pain, which may be a result of the nerve damage.

IL-10 is considered an anti-inflammatory mediator (22) and, in the present study, levels of this cytokine were significantly associated with axonal loss. IL-10 has also been described in the lepromatous pole of leprosy (2). This suggests that patients with PNL these patients may have a form of the disease related to the multibacillary forms, when silent neuritis plays an important role (23).

Cytokines are considered important components of leprosy reactions (7). Type 1 leprosy reactions are characterized by an exacerbated cellular immune response, which can lead to neural damage. In these episodes, high levels of TNF-α, IFN-γ, IL-12, IL-1β, and IL-2 occur (2). Patients with PNL are considered to have fewer reactional episodes when compared to those with other clinical forms of leprosy (9). Only two patients in our group had leprosy reactions at diagnosis, which could explain the lower levels of these cytokines.

Type 2 leprosy reactions are characterized by a systemic process related to immune complexes and high levels of TNF-α, IL-2, IL-4, IL-5, IL-6, and IL-10 (2).Type 2 leprosy reactionsare even more rare that type 1 leprosy reaction in patients with PNL (9)and this may be related to the lower levels of IL-10 found in our sample, suggesting that the immunological profile of patients with PNL is different to that usually found in patients with type 2 leprosy reactions.

Proinflammatory cytokines such as TNF-α and IL-1β can directly stimulate and sensitize Aδ fibers and C-type fibers (17). The spontaneous discharge of these fibers has already been related to neuropathic pain (24). The majority of our cohort did not have neural pain at diagnosis, which could explain the lower levels of these cytokines when compared to others.

Cytokines are produced as a response of the immune system to antigens (7) In PNL the *M. leprae* antigens are confined within the Schwann cell in the peripheral nerve and this may explain why the cytokines usually related to leprosy and leprosy reactions are not found in high levels in the serum of these patients. Furthermore, patients with PNL may present with a wide variety of clinical phenotypes. As the cytokine profile appears to be different from other clinical forms of leprosy, this may suggest that PNL is a different form of the disease that cannot be classified in the classical Ridley–Jopling classification system.

This is one of the first studies to address the cytokine profile in patients with PNL. Although the number of patients of the sample is a limitation of the present study, we believe that the results could provide new information and raise new questions about the immunological pattern and pathophysiology of PNL.

## Data availability statement

The original contributions presented in the study are included in the article/supplementary material. Further inquiries can be directed to the corresponding author.

## Ethics statement

The studies involving humans were approved by Fundação Oswaldo Cruz Ethics in Research Committee. The studies were conducted in accordance with the local legislation and institutional requirements. The participants provided their written informed consent to participate in this study.

## Author contributions

IP: Conceptualization, Data curation, Formal Analysis, Investigation, Writing – original draft, Writing – review & editing. DA: Conceptualization, Data curation, Formal Analysis, Investigation, Methodology, Writing – review & editing. RP: Conceptualization, Investigation, Writing – review & editing. JV: Data curation, Investigation, Writing – review & editing. CS: Investigation, Writing – review & editing. LA: Investigation, Writing – review & editing. LC: Investigation, Writing – review & editing. MH: Conceptualization, Data curation, Formal Analysis, Writing – review & editing. ES: Conceptualization, Investigation, Writing – review & editing. MJ: Conceptualization, Investigation, Supervision, Writing – review & editing.
